# Risk factors for long-term analgesic prescription after hip or knee arthroplasty for osteoarthritis: results of a multivariable regression analysis based on health insurance claims data from 137,875 patients with postoperative inpatient rehabilitation

**DOI:** 10.1186/s12891-026-10465-0

**Published:** 2026-09-17

**Authors:** Caroline Schmuker, Kristin Kieselbach, Vanessa Kaiser, Urs A. Fichtner, Christian Günster

**Affiliations:** 1 Institute of General Practice and Family Medicine, Jena University Hospital, Friedrich Schiller University Jena, Bachstraße 18, Jena, 07743 Germany; 2 AOK Research Institute, Rosenthaler Str. 31, Berlin, 10178 Germany; 3 Interdisciplinary Pain Center, Medical Center University Freiburg, Lehener Str. 86, Freiburg, 79106 Germany; 4 Institute of Medical Biometry and Statistics, Medical Faculty and Medical Center, Section of Health Care Research and Rehabilitation Research, University of Freiburg, Hugstetter Str. 49, Freiburg, 79106 Germany

**Keywords:** Real-world evidence, Pain medication, Joint replacement, Arthroplasty, Rehabilitation, Billing data, Predictors

## Abstract

**Background:**

Continued analgesic prescription after elective total hip arthroplasty (THA) or total knee arthroplasty (TKA) indicates persistent pain and increases the risk of adverse drug reactions, particularly in older patients or those with chronic conditions. The aim of this study was to identify independent risk factors for long-term analgesic prescription in patients who have undergone arthroplasty followed by inpatient orthopaedic rehabilitation.

**Methods:**

The study is based on nationwide claims data from Germany’s largest statutory health insurance provider (AOK) for the period from 2020 to 2024. Potential risk factors included demographic characteristics as well as factors related to the hospital stay/surgery and outpatient treatment in the 12 months prior to surgery. Adjusted odds ratios (OR) were calculated using a multivariable logistic regression model. In addition, we examined the extent to which model performance, measured by the area under the curve (AUC), improved when an additional variable category was included.

**Results:**

The strongest association with long-term analgesic prescription after arthroplasty was observed for intensive use of analgesics in the year prior to surgery, which showed a more than sixfold increase in the odds (THA: OR 6.7; TKA: OR 7.5). A higher care dependency level, substance use disorders, a high BMI (≥ 40 kg/m^2^), and various pain conditions showed significant associations, with adjusted ORs of up to 2.3 in both indications. Including risk factors from the 12-month pre-observation period into the regression model enhanced model performance strongly.

**Conclusion:**

The results of this observational study based on a large nationwide database may provide valuable clinical insights and establish a robust framework for future confirmatory trials.

**Trial registration:**

Not applicable. This study is a retrospective analysis of routinely collected billing data and does not involve health interventions.

**Supplementary Information:**

The online version contains supplementary material available at https://doi.org/10.1186/s12891-026-10465-0.

## Background

Arthroplasty is an established treatment option for advanced osteoarthritis when conservative treatment measures have proven ineffective. Indication criteria for elective total hip arthroplasty (THA) and knee arthroplasty (TKA) include persistent joint pain combined with substantial limitation of mobility and significant pain-related suffering [1, 2]. Although arthroplasty is effective in relieving pain for most patients, a subgroup of patients continues to experience persistent pain following this procedure [3, 4]. Given the elective nature of total joint arthroplasty and the inherent surgical risks involved, it is critical that patients maintain realistic expectations regarding the possibility of suboptimal clinical outcomes or incomplete symptom resolution [5, 6]. This could lead to an ongoing need for analgesics.

A recent umbrella review identified a number of risk factors for persistent pain after arthroplasty, including race, opioid use, preoperative function and pain, neuropathic pain symptoms, pain catastrophizing, anxiety, fear of movement, social support, mental health, or coping strategies [7]. In Germany, most patients undergo around three weeks of inpatient orthopaedic rehabilitation after surgery. The programme aims to reduce pain and enhance joint function and mobility through evidence-based, personalized therapeutic interventions [8]. The present analysis is based on a research project that focuses on quality assurance in medical rehabilitation [9]. The continued long-term prescription of pain medication was identified in this project as an undesirable long-term outcome of rehabilitation following joint replacement surgery. Beyond that, long-term use of analgesics after arthroplasty has public health implications, as it is associated with increased medical care needs, follow-up costs, and potential risks of medication-related morbidity especially in the elderly [10–13].

However, studies on the long-term prescription of pain medications in patients following elective hip or knee arthroplasty remain scarce [14, 15]. International studies have mainly focused on postoperative opioid prescriptions [16–19], while non-opioid analgesics have received less attention. The present study aims to contribute to this research gap by examining both opioid and non-opioid analgesic prescriptions after THA and TKA. All relevant classes of analgesic medication are considered, with a focus on sustained prescriptions at higher volumes. Potential risk factors for long-term analgesic prescription were collected from a nationwide database and assigned to four categories: demographics, inpatient stay (arthroplasty), 12-month pre-observation period and process factors. This data is based on the International Classification of Diseases (ICD-10), operation and procedure codes (OPS), and the Anatomical Therapeutic Chemical (ATC) classification system for prescriptions. Our study addresses the prevalence of these risk factors, their association with long-term use of analgesics, and the influence of each variable category (e.g., demographics, inpatient stay [arthroplasty]) on the improvement of model fit.

## Methods

### Data sources

The study is retrospective and uses nationwide claims data from AOK, Germany's largest statutory health insurance provider. AOK covers more than a third of Germany's population. The dataset spanned from 2020 to 2024, with arthroplasty occurring between 2021 and 2023. The years 2020 and 2024 were designated as the pre- and post-observation periods, to ensure that all relevant pre-existing risk factors and long-term prescriptions were available for analysis. The investigators had full access to the database, and were therefore able to independently select the study population and risk factors. The reporting of this study conforms to the RECORD (REporting of studies Conducted using Observational Routinely-collected health Data) guidelines.

### Study population

The study population included all patients with primary THA or TKA due to osteoarthritis, followed by a postoperative inpatient rehabilitation within six weeks after hospital discharge, covered by the AOK health insurance fund (Table 1). To ensure the inclusion of primary arthroplasty only, patients with implantations, revisions, or removals on the same joint in the last two years prior to index arthroplasty were excluded. Patients with malignant neoplasms as a comorbid condition in addition to joint replacement were excluded in order to reduce risk of capturing analgesic prescriptions due to these conditions.

**Table 1:** Codes and criteria used to identify patients with elective hip and knee arthroplasty followed by inpatient rehabilitation

Procedures and diagnoses	Codes (OPS/ICD-10)	Rehabilitation
Hip Arthroplasty	OPS: 5–820.0, 5–820.8, 5–820.9, 5–820.x	Postoperative IRF stay within 42 days of discharge from hospital
with discharge diagnosis	ICD-10: M05-M08, M16, M87	
Knee Arthroplasty	OPS: 5–822.0, 5–822.g, 5–822.h, 5–822.j, 5–822.k	
with discharge diagnosis	ICD-10: M05-M08, M17, M87	

The fifth digit of OPS code 5–822/5–820 specifies the prosthesis type. The extension.x specifies "other" or "not elsewhere classified" surgical procedures

*OPS* German Operation and Procedure Codes, *ICD* International Classification of Diseases, 10th German Modification, *IRF* Inpatient Rehabilitation Facility

### Outcome measure

Postoperative long-term analgesic prescription was defined as being prescribed at least 20 daily defined doses (DDDs) of analgesics in each of two subsequent quarters (periods of 90 individual days each) after discharge from IRF. In the case of TKA, analgesic prescriptions between days 1 and 28 after discharge from rehabilitation were not taken into account as TKA patients tend to have a greater short-term need for analgesics than THA patients. Analgesic medication included anti-inflammatory and anti-rheumatic drugs (ATC M01), opioids (ATC N02A), and other analgesics and antipyretics (ATC N02B). Over-the-counter pain medication such as non-prescription strength Ibuprofen, is not recorded in claims data. The outcome definition was adopted from Maneck et al. [20] and tailored to pain management following joint replacement. Its clinical relevance and validity was confirmed by medical experts as part of the previously mentioned research project [9].

### Potential risk factors

Risk factors potentially related to long-term analgesic prescription were identified and defined based on literature research and insights gained from structured discussions with medical experts. Variables were divided into four categories: demographics, inpatient hospital stay (arthroplasty), pre-observation period (12 months), and process factors.

Demographic factors included age, biological sex, and the level of long-term care need. In Germany, care levels are categorized on a scale from 1 to 5, representing the progression from slight impairment of independence to the most severe impairment requiring specialized care.

Factors related to "inpatient stay (arthroplasty)" included comorbidities according to the Elixhauser Comorbidity Index and its disease definition by ICD-Codes [21]. However, malignant neoplasms were not included, as these were exclusion criteria. The body mass index (BMI) was included for the classes 30–34, 35–39, and > = 40 kg/m^2^. Frailty was assessed using the Hospital Frailty Risk Score (HFRS), originally developed by Gilbert et al. [22], which has been applied to German health insurance claims data [23] and successfully validated within the orthopaedic context [24]. The HFRS was calculated for each patient using ICD-10 diagnoses with predefined points per diagnosis based on the German modification [23]. Physical therapy during hospital stay (OPS 8–561), early rehabilitative complex treatments (OPS 8–55), and length of hospital stay (in days) were included. Lastly, the category included severe complications during hospital stay, which encompasses general complications, thromboses or pulmonary embolisms, surgical complications, revisions, or fractures as specified by the Quality Assurance with Routine Data (QSR) standards in Germany [25].

The category "pre-observation period (12 months)" encompassed information on outpatient treatment in the year prior to arthroplasty. Chronic conditions such as joint and bone diseases, mental disorders, and chronic pain conditions were included (corresponding ICD-10 codes are reported in Table S1 in the additional file). In addition, persistent prescriptions for analgesics or physical therapy prior to surgery were taken into account: Long-term analgesic use before surgery is defined as a minimum of 20 DDD in at least 3 of 4 quarters (90-day periods) prior to surgery, while physical therapy required at least one prescription (due to a diagnosis of diseases of the extremities and pelvis) up to 90 days before surgery.

The category "process" included the transition period between hospital discharge and rehabilitation admission (in days), and the length of inpatient rehabilitation stay (in days). As an ecological measure, the general complication rate of the hospital that performed the surgery, was included in the models as a risk factor. In Germany, quality of hospitals is assessed yearly by standardized quality indicators based on claims data following up complications such as revisions or prosthesis removal up to 365 days after surgery [25, 26]. Hospitals were classified according to their standardized complication rate (SMR) as having a low (SMR < 0.9), medium (SMR 0.9–1.1), or high (SMR ≥ 1.2) complication rate. As this is an aggregated measure at hospital level, it only reflects contextual characteristics and not individual patient risk.

### Data analyses

The prevalence of risk factors within the study population was described using frequency distributions for categorical variables. Multivariable logistic regression analyses were performed to assess the associations between risk factors and long-term analgesic prescription for THA and TKA separately. Adjusted odds ratios (ORs) with corresponding 95% confidence intervals (CIs) were calculated. Statistical significance was defined as a two-sided p-value less than 0.05. Multicollinearity was assessed by calculating Variance Inflation Factors (VIF). To account for the hierarchical structure of the data and the potential correlation of results within the same rehabilitation facility, the standard errors were clustered at the institutional level (institution code).

To ascertain the relevance of each variable category, the increase in model quality upon the inclusion of each additional category was examined. Model quality was determined using the area under the curve (AUC) with a 95% CI. Model A included only demographic factors; model B included demographic factors and factors from inpatient hospital stay (arthroplasty); model C included all model B factors, plus factors from the pre-observation period (12 months); and model D included all four categories, including process factors. Model calibration was assessed visually using calibration plots. Proximity of the calibration curve to the 45-degree diagonal ideal line was used as the criterion for a well-calibrated model. Missing values only occurred in the outcome variable: A total of 570 THA patients and 485 TKA patients were excluded from the regression analyses because they did not have continuous insurance coverage until the end of the follow-up period and did not experience the outcome beforehand (known as censoring). To provide additional clinical insights, the data set was stratified into two groups based on patient age: under 74 years and 75 years and older. In addition, two sensitivity analyses were conducted. First, patients who underwent additional musculoskeletal surgery during the follow-up period (180 days after IRF discharge) were excluded, as this can result in analgesic prescriptions that are not attributable to the joint replacement. Secondly, to test the temporal stability of our results, the dataset was stratified by individual calendar years from 2021 to 2023.

## Results

The study population comprised a total of 64,635 THA patients and 73,240 TKA patients. Overall, 17.0% of THA patients and 17.2% of TKA patients received long-term analgesic prescription. The prevalence of the potential risk factors is shown in Table 2. In both indications, around 90% of patients were aged 65 and older. Women accounted for over 65% of arthroplasties. Around 15% of patients were classified as care level 1 through 5 at the time of hospital admission. Common comorbidities included hypertension (over 67%) and hypothyroidism (over 20%). Common conditions treated in outpatient care included chronic pain in the spine (THA: 44%; TKA: 40%) and depression (THA: 22%; TKA: 24%). The proportion of patients receiving continued analgesic treatment prior to arthroplasty was 26.9% among THA patients and 24.4% among TKA patients.

**Table 2:** Frequency of selected risk factors in the study population

Risk factor/variable	Hip arthroplasty	Knee arthroplasty
Patients, n (%)	64,635 (100)	73,240 (100)
Demographics		
Age, n (%)		
below 64	6144 (9.5)	7477 (10.2)
65 to 69	14,081 (21.8)	16,947 (23.1)
70 to 74	15,938 (24.7)	19,721 (26.9)
75 to 79	12,659 (19.6)	14,945 (20.4)
80 to 84	11,980 (18.5)	11,427 (15.6)
85 and over	3833 (5.9)	2723 (3.7)
Gender, n (%)		
Male	22,547 (34.9)	24,839 (33.9)
Female	42,088 (65.1)	48,401 (66.1)
Nursing care level, n (%)		
No care level	54,049 (83.6)	62,236 (85.0)
Care level 1	3958 (6.1)	3,845 (5.2)
Care level 2	5590 (8.6)	5896 (8.1)
Care level 3 and higher	1038 (1.6)	1263 (1.7)
Inpatient stay (arthroplasty)		
Comorbidities, n (%)		
BMI 30–34 kg/m2	6089 (9.4)	8989 (12.3)
BMI 35–39 kg/m2	3123 (4.8)	5604 (7.7)
BMI 40 plus kg/m2	1484 (2.3)	3138 (4.3)
Depression	2920 (4.5)	3889 (5.3)
Rheumatic disease	1694 (2.6)	2206 (3.0)
Water and electrolyte and acid–base imbalances	5296 (8.2)	5059 (6.9)
Other neurological disorders	955 (1.5)	1305 (1.8)
Chronic lung disease	5325 (8.2)	6380 (8.7)
Diabetes without complications	10,203 (15.8)	13,851 (18.9)
Diabetes with complications	1011 (1.6)	1308 (1.8)
Hypothyroidism	12,950 (20.0)	16,110 (22.0)
Kidney failure/insufficiency	5420 (8.4)	5664 (7.7)
Congestive heart disease	3010 (4.7)	3063 (4.2)
Cardiac arrhythmia	8399 (13.0)	8970 (12.2)
Heart valve disease	1544 (2.4)	1539 (2.1)
Peripheral vascular disease	1945 (3.0)	1717 (2.3)
Hypertension without complications	43,558 (67.4)	52,028 (71.0)
High blood pressure with complications	1632 (2.5)	1739 (2.4)
Hospital Frailty Risk Score (HFRS), n (%)		
Low risk (< 5)	63,956 (98.9)	72,769 (99.4)
Intermediate risk (5 to 15)	679 (1.1)	470 (0.6)
High risk (> 15)	0	1 (0.0)
Therapies, n (%)		
Early rehabilitation complex treatment	355 (0.5)	239 (0.3)
Function-oriented physical therapy	10,727 (16.6)	11,899 (16.2)
Complications, n (%)		
Revision	156 (0.2)	49 (0.1)
Surgical complication	479 (0.7)	434 (0.6)
Femur fracture	742 (1.1)	
General complication	858 (1.3)	755 (1.0)
Thrombosis/pulmonary embolism	135 (0.2)	329 (0.4)
Days in hospital, n (%)		
< 8	30,199 (46.7)	34,870 (47.6)
8 and 9	22,224 (34.4)	26,030 (35.5)
> 9	12,212 (18.9)	12,340 (16.8)
Pre-observation period (12 months)		
Outpatient ICD-diagnoses, n (%)		
Osteoporosis	8495 (13.1)	8714 (11.9)
Atherosclerosis	6886 (10.7)	6967 (9.5)
Segmental functional disorders	7266 (11.2)	8284 (11.3)
Anxiety disorders	4301 (6.7)	5364 (7.3)
Depression	14,386 (22.3)	18,054 (24.7)
Somatoform disorders	10,382 (16.1)	12,408 (16.9)
Sleep disorder	8138 (12.6)	11,071 (15.1)
Other neurotic disorders	1133 (1.8)	1361 (1.9)
Gait and mobility disorders	7427 (11.5)	7826 (10.7)
Pain: psychogenic	1128 (1.7)	1563 (2.1)
Headache and migraine	3661 (5.7)	4929 (6.7)
Pain: Abdomen	1713 (2.7)	1943 (2.7)
Pain: Extremities	1419 (2.2)	1491 (2.0)
Pain: Spine	28,536 (44.1)	29,609 (40.4)
Other pain (not classified)	14,980 (23.2)	17,767 (24.3)
Prescriptions prior to surgery, n (%)		
Received long-term analgesic	17,410 (26.9)	17,868 (24.4)
Received physiotherapy	7314 (11.3)	6406 (8.7)
Process		
Days between hospital discharge and rehabilitation, n (%)		
< 3	46,513 (72.0)	52,916 (72.3)
3 to 7	9,495 (14.7)	11,112 (15.2)
> 7	8627 (13.3)	9212 (12.6)
Days in inpatient rehabilitation, n (%)		
< 8	1251 (1.9)	1155 (1.6)
8 to 21	57,277 (88.6)	64,804 (88.5)
> 21	6107 (9.4)	7281 (9.9)
Standardized complication rate (hospital), n (%)		
Low (< 0.9)	22,603 (35.0)	28,531 (39.0)
Medium (0.9 to 1.1)	20,473 (31.7)	18,553 (25.3)
Higher (> 1.1)	21,559 (33.4)	26,156 (35.7)

ICD-10 diagnoses with a prevalence ≤ 1 percent are not presented. Outpatient ICD-10 diagnosis was defined as present if recorded in the database in at least 2 of 4 quarters (90d-period). Long-term analgesic use before surgery is defined as a minimum of 20 DDD of analgesic prescription in at least 3 of 4 quarters (90-day periods) in 365 days prior to surgery. Physical therapy is defined at least 1 prescription (Diseases of the extremities and pelvis) up to 90 days before surgery. BMI, Body Mass Index

### Association with long-term analgesic prescription after arthroplasty

**Fig. 1:**
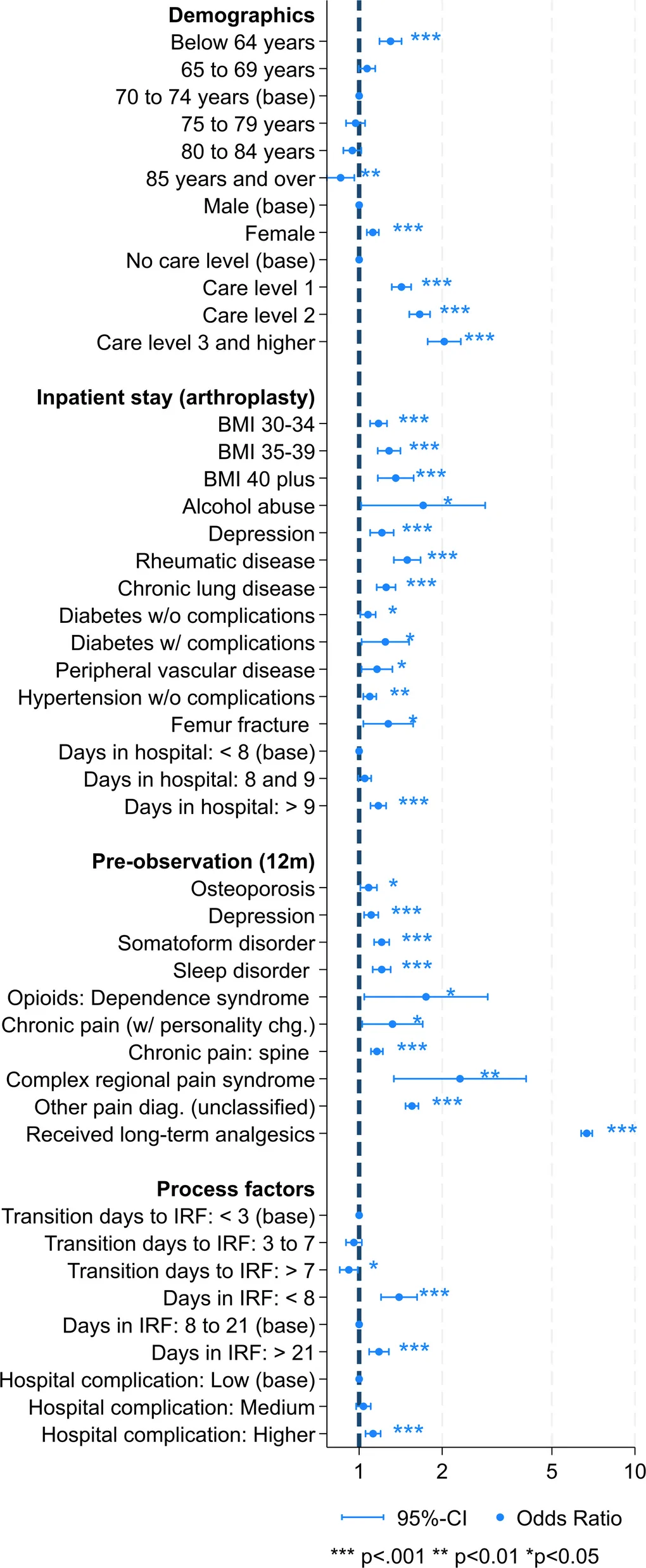
Risk factors for postoperative long-term analgesic prescription after hip arthroplasty. Only variables with statistically significant odds ratios are visualized, except for categorical variables, where all categories are displayed for completeness. The points represent the odds ratio (OR) estimates, and the bars indicate the 95% confidence intervals (CIs). A vertical dashed line at OR = 1 represents the null effect. Detailed numerical estimates for all variables, including non-significant risk factors, are available in Table S2. ICD-10 diagnosis labels have been abbreviated by the author for more clarity. IRF, Inpatient Rehabilitation Facility

**Fig. 2:**
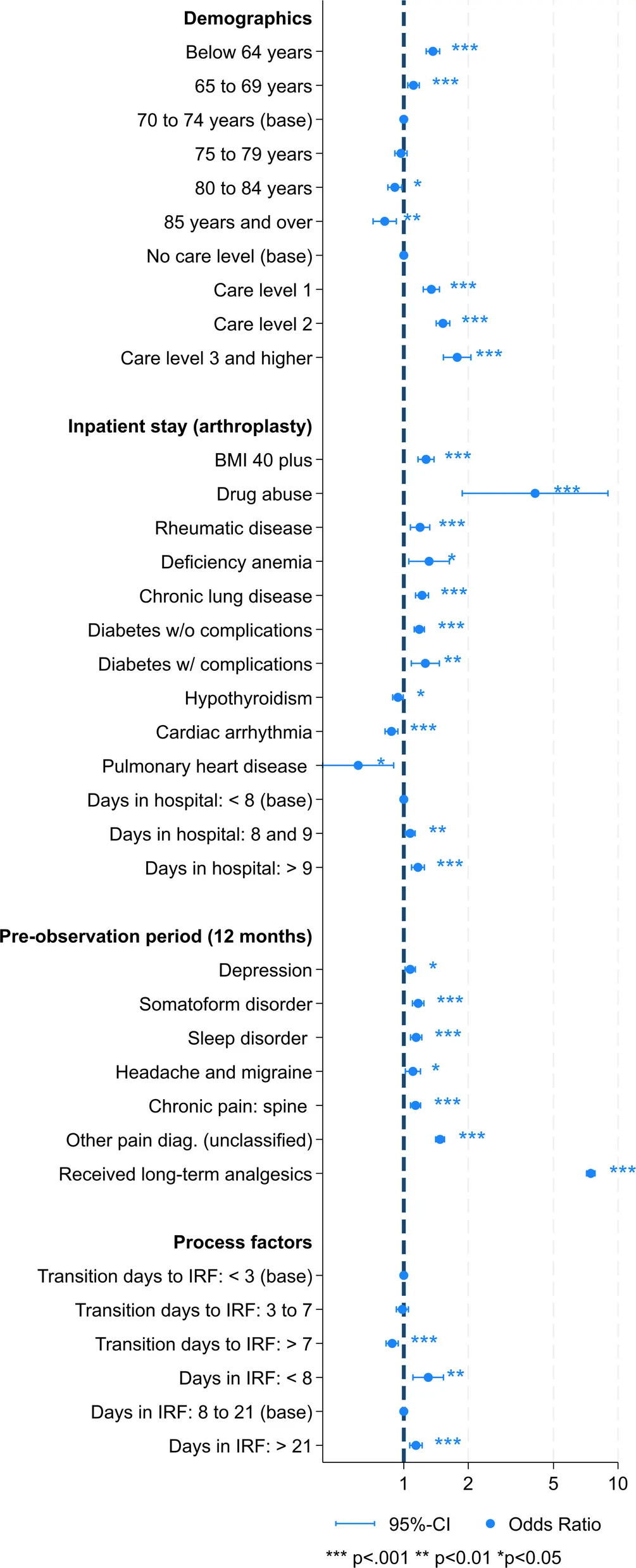
Risk factors for postoperative long-term analgesic prescription after knee arthroplasty. Only variables with statistically significant odds ratios are visualized, except for categorical variables, where all categories are displayed for completeness. The points represent the odds ratio (OR) estimates, and the bars indicate the 95% confidence intervals (CIs). A vertical dashed line at OR = 1 represents the null effect. Detailed numerical estimates for all variables, including non-significant risk factors, are available in Table S2. ICD-10 diagnosis labels have been abbreviated by the author for more clarity. IRF, Inpatient Rehabilitation Facility

Multivariable regression analysis showed a number of significant associations with a few differences between THA (Fig. 1) and TKA (Fig. 2). Regression coefficients of all variables, including nonsignificant results, are available in the additional file (Table S2). VIF was highest in the variables gender and hypertension (VIF = 3). With an average VIF of 1.2 in each indication, multicollinearity was not considered as problematic, despite the high number of independent variables.

Demographics: Younger age (< 65 years) was associated with higher odds of long-term analgesic prescription (THA: OR 1.30; TKA: OR 1.37) compared to the reference group (70–74 years), whereas very old age (≥ 85 years) was associated with lower odds (THA: OR 0.86; TKA: OR 0.81). Female gender was significantly associated with the outcome only among THA patients (OR 1.12). In both indications, the odds for long-term analgesic prescription consistently increased with higher levels of care, reaching their maximum among patients with care level 3 or above (THA: OR 2.02; TKA: OR 1.77).

Hospital stay (arthroplasty): While moderate obesity, evaluated separately as BMI 30–34 kg/m^2^ (OR 1.17) and BMI 35–39 kg/m^2^ (OR 1.28), was significantly associated with the outcome only among THA patients, severe obesity (BMI ≥ 40 kg/m^2^) showed an association among THA patients (OR 1.36) and TKA patients (OR 1.27). Alcohol abuse was correlated with the outcome among THA patients (OR 1.71), while drug abuse showed to be highly correlated exclusively among TKA patients (OR 4.1). Rheumatic comorbidity showed a strong association with the outcome in THA patients (OR 1.49) and less pronounced among TKA patients (OR 1.19). Several comorbidities such as chronic lung disease (THA: OR 1.25; TKA: OR 1.22), diabetes with complications (THA: OR 1.24; TKA: OR 1.26), diabetes without complications (THA: OR 1.08; TKA: OR 1.18), and depression (THA: OR 1.21) showed significant but moderate associations. Complications during hospital stay were not significantly correlated, except for a femur fracture among THA patients (OR 1.27). A length of hospital stay over 10 days was linked to the outcome in both THA and TKA patients (THA: OR 1.17; TKA: OR 1.16). Some comorbidities showed significant inverse associations among TKA patients (e.g., hypothyroidism [OR 0.94], cardiac arrhythmia [OR 0.88], and pulmonary circulation disorders [OR 0.61]), but not among THA patients.

Pre-observational period (12 months): The association between extensive pain medication (≥ 20 DDD) in the year prior to joint arthroplasty and long-term analgesic prescription after arthroplasty was found to be particularly strong (THA: OR 6.69; TKA: OR 7.46). Some pain-related ICD-10 diagnoses from outpatient treatment, such as “pain, unspecified” (THA: OR 1.55; TKA: OR 1.48) and dorsalgia (THA: OR 1.16; TKA: OR 1.13), were correlated with the outcome in both indications, while “complex regional pain syndrome” (OR 2.33) and “chronic pain syndrome” (OR 1.31) only were correlated among THA patients. ICD-10 diagnoses for mental disorders such as “opioid dependency” (THA: OR 1.75), somatoform disorders (THA: OR 1.21; TKA: OR 1.17), and depression (THA: OR 1.11;TKA: OR 1.07) showed stronger association among THA patients than among TKA patients. A diagnosis of a sleep disorder showed a significant association in both indications (THA: OR 1.21; TKA: OR 1.14).

Process: A very short rehabilitation stay (< 8 days) and very long rehabilitation stay (> 21 days) were both associated with the outcome (THA: OR 1.40; TKA: OR 1.30 and THA: OR 1.18; TKA: OR 1.14, respectively), while a longer transition period between hospital discharge and start of inpatient rehabilitation (> 7 days) was inversely associated (THA: OR 0.92; TKA: OR 0.88). A higher hospital complication rate (SMR > 1.2) was correlated with long-term analgesic prescription only among THA patients (OR: 1.12).

Subgroup analysis revealed similar overall trends in younger (< 75 years) and older (> = 75 years) patients undergoing joint replacement surgery, with only a few differences (Table S4 and S5, additional file). The reported significant odds ratios were largely consistent when the analyses were stratified by calendar year. Some risk factors in the TKA model, however, did not show stable associations—that is, the direction of the odds ratio changed in a single year: rheumatic disease deficiency, diabetes with complications, and pulmonary heart disease. Furthermore, after excluding patients who underwent additional musculoskeletal procedures during follow-up, the observed odds ratios remained highly consistent (Table S6, additional file).

The discriminative quality of the regression models for THA and TKA increased with increasing model complexity (Model A to D). The strongest improvement was achieved by adding variables from the 12-month pre-observation period, with an increase in AUC value from 0.635 to 0.784 (THA) and from 0.619 to 0.777 (TKA). AUC values for all model sequences are available in Table S3 (Additional file). Calibration plots for the model using all categories demonstrated good agreement between the predicted probabilities and the observed proportions in both indications (Fig. S1 and S2, additional file).

## Discussion

This claims data analysis identified various demographic, outpatient and hospital-related risk factors associated with long-term analgesic prescription in patients with hip or knee arthroplasty for osteoarthritis. The inclusion of ICD-10 diagnoses and analgesic prescription data from the 12-month pre-observation period notably improved the discriminative accuracy of the regression models for both indications.

In our study, a high prescription volume of analgesics in the year prior to surgery as well as various ICD-10 pain-related diagnoses from the 12-month pre-observation period, showed strong associations with long-term analgesic prescription after arthroplasty. These results suggest that a history of chronic pain and extensive use of analgesics prior to surgery might limit the success of rehabilitation after arthroplasty, which is supported by prior research [18, 27]. There was a strong association between higher levels of long-term care need and long-term analgesic prescription, which (to our knowledge) has not been investigated yet. This finding seems plausible as a higher level of care indicates greater impairment of physical functions and independence, which could potentially lead to increased pain or more difficult postoperative pain management [28].

The odds of receiving long-term analgesic prescription were significantly higher among younger patients than in the reference group.. This finding is in line with other studies that have established a link between younger age and an increased risk of prolonged analgesic prescription [14] and opioid use [18, 19].

The influence of comorbidities diagnosed during hospital stay on long-term analgesic prescription is heterogenous. Rheumatic disease, in particular, was strongly associated with the outcome in patients with THA, while other comorbidities showed no or only low associations. An association between rheumatic diseases and chronic pain after THA could not be confirmed in existing reviews [7] and might therefore be subject to further studies. A high BMI (> 40 kg/m^2^) was highly correlated with long-term analgesic prescription in patients with THA and TKA. An elevated BMI is a well-known individual risk factor for a number of complications [29], for self-reported long-term pain [7, 30] and for overall analgesic use [31] after joint replacement. Some comorbidities, such as hypothyroidism, cardiac arrhythmias or pulmonary circulation disorders, were inversely associated with the outcome among TKA patients, which may be explained by the fact that treatment with analgesics, especially NSAIDs (non-steroidal anti-inflammatory drugs), may be contraindicated in patients with multimorbidity, especially in patients with cardiac diseases [32].

With regard to outpatient treatments our study revealed moderate associations between depression/somatoform disorders and the long-term use of analgesics, as well as a strong association between opioid dependency and long-term use of analgesics. The relationship between mental health conditions and chronic pain is widely recognized as being complex and bidirectional [33]. There is some evidence that psychological factors affect rehabilitation and outcomes following elective orthopaedic surgery [34, 35]. Furthermore, sleep disorders (organic cause) also showed a moderate association with the outcome in patients with THA and TKA. However, evidence supporting this finding is limited. In a prospective study based on patients’ self-reports, preoperative sleep quality was not an independent predictor of chronic postoperative pain 12 months after knee and hip arthroplasty [36].

Both very short and very long rehabilitation stays were associated with long-term analgesic prescription. This could indicate inefficient or particularly complex rehabilitation processes. Conversely, a prolonged transition period between hospital and rehabilitation was found to be slightly inversely associated. The longer transition period may reflect a lower urgency or severity of symptoms, indicating an overall better initial state of health. With regard to hospital quality, an above-average complication rate was found to be weakly associated with long-term analgesic prescription among THA patients, but not among TKA patients. To our knowledge these process factors have not yet been investigated.

The strengths of this study include the large cohort size and definition of numerous individual risk factors that are not easy to collect via patient self-reports without risk of recall bias. The inclusion of ICD-10 diagnoses and analgesic prescription data from the 12-month pre-observation period notably improved the discriminative accuracy of the regression models for both indications. In addition, the outcome definition included opioid and non-opioid analgesics, which adds to existing literature.

However, the study also has limitations. Firstly, administrative claims data were primarily collected for billing purposes rather than for research purposes. Certain factors associated with chronic pain, such as ethnic background, individual coping strategies, social support or anxiety [7], were not considered in our study. These unmeasured factors could influence both preoperative and postoperative analgesic prescriptions, potentially introducing bias into the observed associations. Another limiting factor is that the prescriptions could not be linked to a specific diagnosis. Consequently, the extent of joint-specific pain medication prescriptions may be overestimated. We attempted to reduce the risk of outcome misclassification by adjusting for various pain conditions from the ICD catalogue that are not attributable to joint replacement. However, since we considered only a selection of pain conditions, this limitation could not be fully eliminated. Furthermore, our dataset does not contain information on the use of over-the-counter (OTC) medications. Consequently, both preoperative and postoperative medication use are underestimated in our analysis.

Our retrospective study estimated effects which are specific to this dataset and therefore the models may be overfitted. Further studies involving external validation are essential. Due to the exploratory nature of the study and the large number of variables involved, no adjustment was performed for multiple testing, thereby increasing the risk of type I errors (false positives). Marginally significant p-values should therefore be interpreted with caution. Additionally, some of these risk factors (e.g. drug and alcohol abuse) have a very low prevalence, meaning the magnitude and precision of their estimates should also be interpreted with caution.

The associations identified in this study are valid only for the chosen study design. It is unclear to what extent the results are robust to variations, for example different time windows for outcome measurement. It is also unclear to what extent the results can be transferred to arthroplasty patients who did not undergo inpatient rehabilitation. Furthermore, our results may be limited in their generalizability to other countries due to structural and cultural differences in healthcare systems. For instance, inpatient rehabilitation following joint replacement surgery is not as prevalent in many other countries as it is in Germany. Furthermore, there are international differences in prescribing practices. This is well documented in the literature, particularly with regard to the use of opioids [37].

## Conclusions

In order to better understand factors associated with the long-term analgesic prescription following hip or knee arthroplasty, it is important to consider a wide range of individual risk factors, including medical treatments received in the year prior to surgery. Using claims data as “real-world evidence” offers a significant advantage for research, as it captures large-scale patterns in the healthcare system. While these findings are not intended for individual clinical prediction, the identified associations can be valuable in practice by highlighting specific high-risk subgroups and providing hypotheses for further research.

## Supplementary Information

Additional file 1: Supplementary Tables S1–S6 and Figures S1/S2. This file contains six additional tables and two figures supporting the main analysis. Table S1 provides the Coding (ICD-10, German Modification) used to define conditions documented in outpatient treatment. Table S2 shows the full regression results including all evaluated risk factors from the logistic regression analyses for long-term analgesic prescription after hip or knee arthroplasty. Table S3 presents the discriminative quality (AUC values) of logistic regression models A-D. Table S4 provides the full regression results stratified by age groups (age <75 years; >=75 years) for THA patients and Table S5 for TKA patients. Table S6 shows the full regression results of the sensitivity analyses when patients with further surgeries on the musculoskeletal system during follow-up (180 days after rehabilitation) where excluded. Figures S1 and S2 show the calibration plots for the THA and TKA models.

## Data Availability

The dataset supporting the conclusions of this article is not publicly available due to strict legal and data protection restrictions regarding German statutory health insurance data (§ 75b SGB V). Consequently, the authors are not permitted to share the raw data. Requests for data access or replication analyses may be directed to the corresponding author or the relevant regulatory authorities, subject to ethical and legal clearance.

## Abbreviations

ATC: Anatomical Therapeutic Chemical Classification System
AUC: Area under the curve
BMI: Body Mass Index
CI: Confidence Interval
DDD: Daily defined doses
HFRS: Hospital Frailty Risk Score
ICD-10: International Classification of Diseases (ICD-10)
IRF: Inpatient Rehabilitation Facilities
NSAID: Non-steroidal anti-inflammatory drug
OPS: Operation and procedure codes
OR: Odds Ratio
THA: Total hip arthroplasty
TKA: Total knee arthroplasty
SHI: Statutory Health Insurance
VIF: Variance Inflation Factor
