# Supplementary material for: Risk factors for long-term analgesic prescription after hip or knee arthroplasty for osteoarthritis: results of a multivariable regression analysis based on health insurance claims data from 137,875 patients with postoperative inpatient rehabilitation
Source: BMC Musculoskelet Disord. 2026 Sep 17;27:828. doi: 10.1186/s12891-026-10465-0 (PMC13640226; doi:10.1186/s12891-026-10465-0)
Supplement: Supplementary file 1 [file 12891_2026_10465_MOESM1_ESM.docx]

Additional file

Tables S1–S6 and figures S1 and S2.

**Table S1:** Coding (ICD-10, German Modification) used to define conditions documented in outpatient treatment

| Disease | ICD-10 Code |
| --- | --- |
| **Joint and bone disorders** |  |
| Osteoporosis | M80 - M82 |
| Fibromyalgia | M79.9 |
| Atherosclerosis | I70 |
| Segmental functional disorders | M99 |
| Gait and mobility disorders | R26 |
| **Mental disorders** |  |
| Phobic disorders | F40 |
| Other anxiety disorders | F41 |
| Depression | F20.4, F31.1, F31.3 bis F31.5, F32, F33, F34.1, F41.2, F43.2 |
| Somatoform disorders | F45 |
| Opioid dependence | F11.2 |
| Other neurotic disorders | F48 |
| Other personality and behavioral disorders: Development of physical symptoms for psychological reasons | F68.0 |
| Chronic pain syndrome with persistent personality change | F62.8 |
| Consumption of alcohol, tobacco, medicines, or drugs | Z72.0 |
| **Other** |  |
| Sleep disorder | G47 |
| **Pain disorders** |  |
| Complex regional pain syndrome | G90.5 bis G90.7 |
| Headache or migraine | R51, G44, G43 |
| Renal colic | N23 |
| Pain: Abdomen | R10 |
| Pain: Eye | H57.1 |
| Pain: Extremity | M79.6 |
| Pain: Joint | M25.5 |
| Pain: Ear | H92.0 |
| Pain: Neck and thorax | R07 |
| Pain: Spine | M54 |
| Pain: Teeth | K08.88 |
| Pain: Tongue | K14.6 |
| Pain, not classified elsewhere | R52 |

Outpatient ICD-10 diagnosis was defined as present if recorded in the claims database in at least 2 of 4 quarters (90d-period). Official ICD-10 diagnoses labels (ICD-10, G*erman* M*odification*) have been abbreviated by the author.

**Table S2:** Full regression results including all evaluated risk factors from the logistic regression analyses for long-term analgesic prescription after hip or knee arthroplasty

|  | Hip arthroplasty | | Knee arthroplasty | |
| --- | --- | --- | --- | --- |
|  | OR | 95% CI | OR | 95% CI |
| **Demographics** | | | | |
| Age |  |  |  |  |
| below 64 | 1.30*** | [1.18, 1.42] | 1.37*** | [1.27, 1.47] |
| 65 to 69 | 1.07 | [1.00, 1.14] | 1.11*** | [1.04, 1.18] |
| 70 to 74 | (Reference) | | (Reference) | |
| 75 to 79 | 0.97 | [0.90, 1.05] | 0.97 | [0.91, 1.04] |
| 80 to 84 | 0.94 | [0.88, 1.02] | 0.91* | [0.84, 0.98] |
| 85 and over | 0.86** | [0.76, 0.96] | 0.81** | [0.72, 0.92] |
| Gender |  |  |  |  |
| Male | (Reference) | | (Reference) | |
| Female | 1.12*** | [1.07, 1.18] | 1.02 | [0.97, 1.08] |
| Nursing care level |  |  |  |  |
| No care level | (Reference) | | (Reference) | |
| Care level 1 | 1.42*** | [1.31, 1.54] | 1.35*** | [1.23, 1.47] |
| Care level 2 | 1.66*** | [1.52, 1.81] | 1.52*** | [1.42, 1.64] |
| Care level 3 and higher | 2.03*** | [1.77, 2.34] | 1.77*** | [1.53, 2.06] |
| **Inpatient stay (arthroplasty)** | | | | |
| BMI 30-34 kg/m^2^ | 1.18*** | [1.10, 1.26] | 1.04 | [0.97, 1.10] |
| BMI 35-39 kg/m^2^ | 1.28*** | [1.17, 1.41] | 1.01 | [0.92, 1.10] |
| BMI 40+ kg/m^2^ | 1.36*** | [1.17, 1.58] | 1.27*** | [1.17, 1.38] |
| Alcohol abuse | 1.71* | [1.02, 2.86] | 1.09 | [0.61, 1.94] |
| Drug abuse | 1.82 | [0.93, 3.57] | 4.10*** | [1.87, 8.97] |
| Psychosis | 0.59 | [0.34, 1.02] | 0.94 | [0.61, 1.45] |
| Depression | 1.21*** | [1.10, 1.33] | 1.05 | [0.96, 1.14] |
| AIDS/HIV | 2.34 | [0.13, 43.30] | 3.39 | [0.64, 17.85] |
| Rheumatic disease | 1.49*** | [1.34, 1.67] | 1.19*** | [1.07, 1.32] |
| Coagulopathy | 1.10 | [0.88, 1.37] | 0.90 | [0.72, 1.13] |
| Weight loss | 0.94 | [0.72, 1.23] | 0.98 | [0.60, 1.59] |
| Disturbances of water, electrolyte or acid-base balance | 1.07 | [0.98, 1.16] | 1.09 | [1.00, 1.18] |
| Hemorrhagic anemia | 1.01 | [0.59, 1.76] | 0.85 | [0.40, 1.83] |
| Deficiency anemia | 1.16 | [0.94, 1.44] | 1.31* | [1.06, 1.63] |
| Other neurological disease | 1.00 | [0.84, 1.19] | 1.00 | [0.85, 1.17] |
| Chronic lung disease | 1.25*** | [1.16, 1.36] | 1.22*** | [1.14, 1.30] |
| Diabetes without complications | 1.08* | [1.01, 1.15] | 1.18*** | [1.12, 1.25] |
| Diabetes with complications | 1.24* | [1.02, 1.52] | 1.26** | [1.08, 1.47] |
| Hypothyroidism | 1.00 | [0.95, 1.05] | 0.94* | [0.89, 0.99] |
| Kidney failure/insufficiency | 1.04 | [0.95, 1.13] | 1.05 | [0.97, 1.13] |
| Liver disease | 0.92 | [0.69, 1.23] | 1.08 | [0.80, 1.45] |
| Peptic ulcer without bleeding | 2.36 | [0.71, 7.87] | 1.68 | [0.62, 4.56] |
| Congestive heart disease | 1.11 | [0.99, 1.24] | 0.96 | [0.86, 1.07] |
| Cardiac arrhythmia | 0.93 | [0.86, 1.00] | 0.88*** | [0.82, 0.94] |
| Heart valve disease | 0.94 | [0.80, 1.10] | 1.06 | [0.91, 1.24] |
| Pulmonary heart and diseases and of the pulmonary circulation | 0.91 | [0.61, 1.34] | 0.61* | [0.42, 0.90] |
| Peripheral vascular disease | 1.16* | [1.02, 1.32] | 1.03 | [0.89, 1.18] |
| High blood pressure without complications | 1.09** | [1.04, 1.15] | 1.05 | [1.00, 1.11] |
| High blood pressure with complications | 0.98 | [0.84, 1.14] | 1.03 | [0.88, 1.20] |
| Paralysis | 1.02 | [0.67, 1.55] | 1.38 | [0.94, 2.01] |
| Frailty-Score |  |  |  |  |
| low risk (<5) | (Reference) | | (Reference) | |
| intermediate risk (5 to 15) | 0.98 | [0.80, 1.20] | 0.89 | [0.69, 1.15] |
| Early rehabilitation complex treatment | 1.06 | [0.78, 1.44] | 0.82 | [0.56, 1.20] |
| Function-oriented physical therapy | 0.99 | [0.92, 1.05] | 0.94 | [0.88, 1.00] |
| Revision | 0.96 | [0.58, 1.60] | 1.92 | [0.89, 4.13] |
| Surgical complication | 1.09 | [0.84, 1.41] | 1.13 | [0.85, 1.51] |
| Femur fracture | 1.27* | [1.04, 1.57] |  |  |
| General complication | 0.89 | [0.73, 1.10] | 0.83 | [0.66, 1.04] |
| Thrombosis/pulmonary embolism | 0.96 | [0.53, 1.74] | 1.32 | [0.97, 1.79] |
| Days in hospital |  |  |  |  |
| < 8 | (Reference) | | (Reference) | |
| 8 and 9 | 1.05 | [0.99, 1.11] | 1.07** | [1.02, 1.13] |
| > 9 | 1.17*** | [1.10, 1.25] | 1.16*** | [1.09, 1.25] |
| **Pre-observation period (12 months)** | | | | |
| Osteoporosis | 1.08* | [1.01, 1.16] | 1.05 | [0.99, 1.12] |
| Atherosclerosis | 1.02 | [0.95, 1.10] | 1.03 | [0.95, 1.11] |
| Fibromyalgia | 1.00 | [0.54, 1.85] | 1.50 | [0.92, 2.44] |
| Segmental functional disorders | 1.00 | [0.93, 1.07] | 0.99 | [0.93, 1.06] |
| Anxiety disorders | 0.93 | [0.86, 1.02] | 1.05 | [0.98, 1.13] |
| Depression | 1.11*** | [1.04, 1.17] | 1.07* | [1.01, 1.13] |
| Somatoform disorders | 1.21*** | [1.13, 1.29] | 1.17*** | [1.10, 1.24] |
| Sleep disorder | 1.21*** | [1.12, 1.30] | 1.14*** | [1.07, 1.21] |
| Mental and behavioral disorders caused by opioids: Dependence syndrome | 1.75* | [1.04, 2.93] | 1.65 | [0.95, 2.84] |
| Other neurotic disorders | 0.88 | [0.74, 1.05] | 0.99 | [0.85, 1.14] |
| Other personality and behavioral disorders: Development of physical symptoms | 0.79 | [0.36, 1.73] | 1.10 | [0.52, 2.33] |
| Consumption of alcohol, tobacco, medicines, or drugs | 0.97 | [0.54, 1.72] | 1.16 | [0.74, 1.84] |
| Gait and mobility disorders | 1.01 | [0.94, 1.08] | 1.01 | [0.95, 1.08] |
| Chronic pain syndrome with persistent personality change | 1.32* | [1.03, 1.70] | 1.00 | [0.80, 1.25] |
| Pain: psychogenic | 0.99 | [0.85, 1.14] | 1.00 | [0.87, 1.13] |
| Headache and migraine | 0.97 | [0.87, 1.07] | 1.10* | [1.02, 1.20] |
| Renal colic | 1.13 | [0.51, 2.51] | 1.68 | [0.95, 3.00] |
| Pain: abdomen | 1.07 | [0.93, 1.23] | 1.01 | [0.88, 1.16] |
| Pain: extremity | 1.14 | [0.98, 1.31] | 1.13 | [0.98, 1.31] |
| Pain: neck and thorax | 0.95 | [0.73, 1.24] | 0.94 | [0.73, 1.21] |
| Pain: spine | 1.16*** | [1.10, 1.22] | 1.13*** | [1.08, 1.20] |
| Pain: ear | 1.02 | [0.54, 1.93] | 0.89 | [0.54, 1.45] |
| Complex regional pain syndrome | 2.32** | [1.34, 4.03] | 1.09 | [0.68, 1.76] |
| Other pain | 1.55*** | [1.47, 1.64] | 1.48*** | [1.41, 1.55] |
| Prescriptions prior to surgery |  |  |  |  |
| Received long-term analgesic | 6.69*** | [6.38, 7.00] | 7.46*** | [7.13, 7.81] |
| Received physiotherapy | 1.00 | [0.93, 1.08] | 0.98 | [0.91, 1.06] |
| **Process** | | | | |
| Days between hospital discharge and rehabilitation |  |  |  |  |
| < 3 days | (Reference) | | (Reference) | |
| 3 to 7 days | 0.96 | [0.90, 1.02] | 0.99 | [0.92, 1.05] |
| >7 days | 0.92* | [0.85, 0.99] | 0.88*** | [0.83, 0.94] |
| Days in inpatient rehabilitation |  |  |  |  |
| < 8 | 1.40*** | [1.20, 1.62] | 1.30** | [1.10, 1.53] |
| 8 to 21 | (Reference) | | (Reference) | |
| >21 | 1.18*** | [1.09, 1.28] | 1.14*** | [1.07, 1.22] |
| Standardized complication rate (surgery hospital) |  |  |  |  |
| Low (<0.9) | (Reference) | | (Reference) | |
| Medium (0.9 to 1.1) | 1.04 | [0.98, 1.10] | 1.00 | [0.95, 1.06] |
| Higher (>1.1) | 1.12*** | [1.05, 1.20] | 1.04 | [0.99, 1.10] |
|  |  |  |  |  |
| Intercept | 0.04*** | [0.04, 0.05] | 0.06*** | [0.05, 0.06] |
| *** p<.001, ** p<.01, * p<.05 |  |  |  |  |

Long-term analgesic prescription after arthroplasty is defined as a minimum of 20 DDD of analgesic prescription in each of 2 subsequent quarters (90-day periods). Outpatient ICD-10 diagnosis was defined as present if recorded in the database in at least 2 of 4 quarters (90d-period). Long-term analgesic use before surgery is defined as at least 20 DDD of analgesic prescription in at least 3 of 4 quarters (90-day periods) in 365 days prior to surgery. Received physical therapy is defined as at least 1 prescription with diagnosis “Diseases of the extremities and pelvis” up to 90 days before surgery.

**Table S3:** Discriminative quality (AUC values) of logistic regression models A-D for hip and knee arthroplasty

|  | Hip arthroplasty | | Knee arthroplasty | |
| --- | --- | --- | --- | --- |
| Model | AUC | 95% CI | AUC | 95% CI |
| A | 0.604 | [0.598, 0.610] | 0.595 | [0.590, 0.601] |
| B | 0.635 | [0.629, 0.640] | 0.619 | [0.614, 0.625] |
| C | 0.784 | [0.779, 0.789] | 0.777 | [0.773, 0.782] |
| D | 0.785 | [0.780, 0.790] | 0.779 | [0.774, 0.783] |

Model A: demographic risk factors

Model B: demographic and inpatient stay-related risk factors

Model C: demographic, inpatient stay (arthroplasty)-related, and pre-observation period (12 months)-related risk factors

Model D: demographic, inpatient stay (arthroplasty)-related, pre-observation period (12 months)-related, and process-related risk factors

|  |  |
| --- | --- |
| **Fig. S1:** Calibration Plot for full THA-Model (Total Hip Arthroplasty) | **Fig. S2:** Calibration Plot for full TKA-Model (Total Knee Arthroplasty) |

**Table S4:** Subgroup analyses: Full regression results including all evaluated risk factors from the logistic regression analyses for long-term analgesic prescription after **hip arthroplasty** stratified by age groups

|  | Age < 75 years  (n=36136) | | Age >=75 Years  (n=28472) | |
| --- | --- | --- | --- | --- |
|  | OR | 95% CI | OR | 95% CI |
| **Demographics** | | | | |
| Gender |  |  |  |  |
| Male | (Reference) | | (Reference) | |
| Female | 1.08* | [1.01, 1.16] | 1.15*** | [1.06, 1.25] |
| Nursing care level |  |  |  |  |
| No care level | (Reference) | | (Reference) | |
| Care level 1 | 1.47*** | [1.29, 1.68] | 1.37*** | [1.23, 1.52] |
| Care level 2 | 1.81*** | [1.59, 2.07] | 1.55*** | [1.40, 1.72] |
| Care level 3 and higher | 2.56*** | [2.07, 3.17] | 1.73*** | [1.44, 2.07] |
| **Inpatient stay (arthroplasty)** | | | | |
| BMI 30-34 kg/m^2^ | 1.19*** | [1.08, 1.30] | 1.17* | [1.03, 1.33] |
| BMI 35-39 kg/m^2^ | 1.26*** | [1.12, 1.41] | 1.41*** | [1.17, 1.71] |
| BMI 40+ kg/m^2^ | 1.41*** | [1.20, 1.66] | 1.31 | [0.93, 1.84] |
| Alcohol abuse | 2.20** | [1.27, 3.81] | 0.26 | [0.02, 2.85] |
| Drug abuse | 2.62* | [1.15, 5.97] | 0.75 | [0.21, 2.70] |
| Psychosis | 0.62 | [0.34, 1.14] | 0.54 | [0.09, 3.23] |
| Depression | 1.23*** | [1.09, 1.38] | 1.22* | [1.04, 1.42] |
| AIDS/HIV | 2.40 | [0.09, 63.58] | 1,00 |  |
| Rheumatic disease | 1.70*** | [1.46, 1.98] | 1.28* | [1.05, 1.55] |
| Coagulopathy | 1.20 | [0.89, 1.63] | 0.96 | [0.68, 1.34] |
| Weight loss | 1.07 | [0.68, 1.69] | 0.85 | [0.59, 1.23] |
| Disturbances of water, electrolyte or acid-base balance | 1.08 | [0.96, 1.21] | 1.04 | [0.93, 1.17] |
| Hemorrhagic anemia | 1.50 | [0.76, 2.96] | 0.60 | [0.22, 1.67] |
| Deficiency anemia | 1.19 | [0.84, 1.69] | 1.14 | [0.88, 1.49] |
| Other neurological disease | 1.14 | [0.91, 1.43] | 0.85 | [0.64, 1.13] |
| Chronic lung disease | 1.36*** | [1.23, 1.50] | 1.12 | [0.99, 1.26] |
| Diabetes without complications | 1.12* | [1.02, 1.22] | 1.02 | [0.92, 1.12] |
| Diabetes with complications | 1.26 | [0.98, 1.63] | 1.25 | [0.99, 1.58] |
| Hypothyroidism | 1.03 | [0.95, 1.11] | 0.96 | [0.89, 1.04] |
| Kidney failure/insufficiency | 0.97 | [0.84, 1.12] | 1.07 | [0.96, 1.20] |
| Liver disease | 0.93 | [0.65, 1.34] | 0.97 | [0.62, 1.54] |
| Peptic ulcer without bleeding | 6.17 | [0.28, 135.19] | 1.46 | [0.28, 7.55] |
| Congestive heart disease | 1.14 | [0.97, 1.35] | 1.07 | [0.92, 1.25] |
| Cardiac arrhythmia | 0.93 | [0.83, 1.05] | 0.91 | [0.82, 1.00] |
| Heart valve disease | 1.01 | [0.81, 1.27] | 0.90 | [0.71, 1.12] |
| Pulmonary heart and diseases and of the pulmonary circulation | 1.53 | [0.77, 3.03] | 0.70 | [0.44, 1.12] |
| Peripheral vascular disease | 1.18 | [0.96, 1.45] | 1.14 | [0.96, 1.37] |
| High blood pressure without complications | 1.09** | [1.02, 1.17] | 1.05 | [0.96, 1.14] |
| High blood pressure with complications | 0.93 | [0.71, 1.23] | 0.97 | [0.80, 1.18] |
| Paralysis | 1.14 | [0.69, 1.88] | 0.85 | [0.39, 1.83] |
| Frailty-Score |  |  |  |  |
| low risk (<5) | (Reference) | | (Reference) | |
| intermediate risk (5 to 15) | 0.79 | [0.54, 1.14] | 1.11 | [0.87, 1.42] |
| Early rehabilitation complex treatment | 0.97 | [0.53, 1.77] | 1.05 | [0.74, 1.48] |
| Function-oriented physical therapy | 1.04 | [0.95, 1.13] | 0.93 | [0.85, 1.02] |
| Revision | 0.88 | [0.47, 1.64] | 1.12 | [0.51, 2.44] |
| Surgical complication | 1.04 | [0.75, 1.45] | 1.19 | [0.79, 1.80] |
| Femur fracture | 1.38* | [1.04, 1.83] | 1.16 | [0.86, .57] |
| General complication | 0.74 | [0.54, 1.01] | 1.02 | [0.78, 1.33] |
| Thrombosis/pulmonary embolism | 0.47 | [0.17, 1.34] | 1.45 | [0.75, 2.79] |
| Days in hospital |  |  |  |  |
| < 8 | (Reference) | | (Reference) | |
| 8 and 9 | 1.03 | [0.96, 1.11] | 1.07 | [0.98, 1.16] |
| > 9 | 1.11* | [1.01, 1.22] | 1.23*** | [1.12, 1.36] |
| **Pre-observation period (12 months)** | | | | |
| Osteoporosis | 1.09 | [0.99, 1.21] | 1.06 | [0.96, 1.17] |
| Atherosclerosis | 1.02 | [0.92, 1.13] | 1.00 | [0.90, 1.11] |
| Fibromyalgia | 1.42 | [0.69, 2.96] | 0.37 | [0.08, 1.74] |
| Segmental functional disorders | 0.98 | [0.89, 1.08] | 1.02 | [0.92, 1.14] |
| Anxiety disorders | 0.90 | [0.81, 1.01] | 0.99 | [0.86, 1.15] |
| Depression | 1.14** | [1.05, 1.24] | 1.08 | [0.99, 1.19] |
| Somatoform disorders | 1.22*** | [1.12, 1.32] | 1.22*** | [1.10, 1.34] |
| Sleep disorder | 1.20*** | [1.09, 1.31] | 1.22*** | [1.10, 1.34] |
| Mental and behavioral disorders caused by opioids: Dependence syndrome | 1.49 | [0.81, 2.77] | 6.56** | [1.96, 21.93] |
| Other neurotic disorders | 0.95 | [0.75, 1.20] | 0.79 | [0.59, 1.05] |
| Other personality and behavioral disorders: Development of physical symptoms | 1.35 | [0.58, 3.13] | 0.21 | [0.02, 2.04] |
| Consumption of alcohol, tobacco, medicines, or drugs | 1.27 | [0.68, 2.36] | 0.18 | [0.02, 1.90] |
| Gait and mobility disorders | 1.01 | [0.89, 1.14] | 0.99 | [0.91, 1.08] |
| Chronic pain syndrome with persistent personality change | 1.45* | [1.08, 1.94] | 1.12 | [0.72, 1.73] |
| Pain: psychogenic | 0.99 | [0.82, 1.20] | 0.99 | [0.78, 1.27] |
| Headache and migraine | 1.01 | [0.89, 1.15] | 0.91 | [0.78, 1.07] |
| Renal colic | 1.19 | [0.45, 3.15] | 1.05 | [0.21, 5.14] |
| Pain: abdomen | 1.13 | [0.94, 1.35] | 0.98 | [0.79, 1.20] |
| Pain: extremity | 1.01 | [0.83, 1.24] | 1.33** | [1.08, 1.64] |
| Pain: neck and thorax | 0.78 | [0.53, 1.16] | 1.15 | [0.78, 1.69] |
| Pain: spine | 1.14*** | [1.07, 1.22] | 1.18*** | [1.10, 1.27] |
| Pain: ear | 1.26 | [0.53, 3.02] | 0.85 | [0.34, 2.17] |
| Complex regional pain syndrome | 2.63* | [1.20, 5.78] | 2.04 | [0.89, 4.68] |
| Other pain | 1.54*** | [1.43, 1.66] | 1.57*** | [1.46, 1.70] |
| Prescriptions prior to surgery | 7.15*** | [6.70, 7.63] | 6.20*** | [5.81, 6.62] |
| Received long-term analgesic | 0.97 | [0.88, 1.06] | 1.06 | [0.94, 1.18] |
| Received physiotherapy | 1.09 | [0.99, 1.21] | 1.06 | [0.96, 1.17] |
| **Process** | | | | |
| Days between hospital discharge and rehabilitation |  |  |  |  |
| < 3 days | (Reference) | | (Reference) | |
| 3 to 7 days | 0.95 | [0.87, 1.04] | 0.99 | [0.89, 1.11] |
| >7 days | 0.93 | [0.85, 1.02] | 0.93 | [0.83, 1.04] |
| Days in inpatient rehabilitation |  |  |  |  |
| < 8 | 1.44*** | [1.16, 1.77] | 1.37** | [1.11, 1.70] |
| 8 to 21 | (Reference) | | (Reference) | |
| >21 | 1.13* | [1.03, 1.25] | 1.26*** | [1.12, 1.43] |
| Standardized complication rate (surgery hospital) |  |  |  |  |
| Low (<0.9) | (Reference) | | (Reference) | |
| Medium (0.9 to 1.1) | 1.04 | [0.96, 1.14] | 1.02 | [0.94, 1.12] |
| Higher (>1.1) | 1.16*** | [1.07, 1.26] | 1.07 | [0.98, 1.17] |
|  |  |  |  |  |
| Intercept | 0.05*** | [0.04, 0.05] | 0.05*** | [0.04, 0.05] |
| *** p<.001, ** p<.01, * p<.05 |  |  |  |  |

Age was excluded as a risk factor because the stratification itself accounts for the age effect. Long-term analgesic prescription after arthroplasty is defined as a minimum of 20 DDD of analgesic prescription in each of 2 subsequent quarters (90-day periods). Outpatient ICD-10 diagnosis was defined as present if recorded in the database in at least 2 of 4 quarters (90d-period). Long-term analgesic use before surgery is defined as a minimum of 20 DDD of analgesic prescription in at least 3 of 4 quarters (90-day periods) in 365 days prior to surgery. Physical therapy is defined at least 1 prescription (Diseases of the extremities and pelvis) up to 90 days before surgery. BMI, Body Mass Index

**Table S5:** Subgroup analyses: Full regression results including all evaluated risk factors from the logistic regression analyses for long-term analgesic prescription after **knee arthroplasty** stratified by age groups

|  | Age < 75 years  (n=44145) | | Age >=75 years  (n=29095) | |
| --- | --- | --- | --- | --- |
|  | OR | 95% CI | OR | 95% CI |
| **Demographics** | | | | |
| Gender |  |  |  |  |
| Male | (Reference) | | (Reference) | |
| Female | 1.01 | [0.95, 1.08] | 1.04 | [0.94, 1.14] |
| Nursing care level |  |  |  |  |
| No care level | (Reference) | | (Reference) | |
| Care level 1 | 1.39*** | [1.22, 1.58] | 1.28*** | [1.14, 1.45] |
| Care level 2 | 1.52*** | [1.38, 1.69] | 1.50*** | [1.37, 1.66] |
| Care level 3 and higher | 1.66*** | [1.36, 2.03] | 1.89*** | [1.54, 2.32] |
| **Inpatient stay (arthroplasty)** | | | | |
| BMI 30-34 kg/m^2^ | 1.03 | [0.95, 1.12] | 1.07 | [0.97, 1.19] |
| BMI 35-39 kg/m^2^ | 1.05 | [0.95, 1.16] | 0.96 | [0.82, 1.12] |
| BMI 40+ kg/m^2^ | 1.29*** | [1.17, 1.42] | 1.55** | [1.19, 2.03] |
| Alcohol abuse | 0.99 | [0.56, 1.73] | 2.85 | [0.64, 12.68] |
| Drug abuse | 5.51*** | [2.21, 13.73] | 2.30 | [0.74, 7.21] |
| Psychosis | 1.07 | [0.68, 1.68] | 0.34 | [0.04, 2.85] |
| Depression | 1.03 | [0.92, 1.15] | 1.17 | [0.99, 1.39] |
| AIDS/HIV | 1,00 |  | 1,00 |  |
| Rheumatic disease | 1.24*** | [1.10, 1.39] | 1.15 | [0.93, 1.42] |
| Coagulopathy | 0.99 | [0.75, 1.31] | 0.79 | [0.50, 1.24] |
| Weight loss | 0.96 | [0.41, 2.22] | 0.96 | [0.51, 1.81] |
| Disturbances of water, electrolyte or acid-base balance | 1.14* | [1.02, 1.26] | 1.03 | [0.91, 1.16] |
| Hemorrhagic anemia | 0.63 | [0.19, 2.12] | 1.05 | [0.45, 2.46] |
| Deficiency anemia | 1.68*** | [1.23, 2.29] | 0.95 | [0.68, 1.33] |
| Other neurological disease | 1.11 | [0.91, 1.35] | 0.85 | [0.64, 1.13] |
| Chronic lung disease | 1.23*** | [1.13, 1.34] | 1.21** | [1.07, 1.37] |
| Diabetes without complications | 1.26*** | [1.17, 1.34] | 1.07 | [0.98, 1.16] |
| Diabetes with complications | 1.39** | [1.12, 1.71] | 1.10 | [0.87, 1.38] |
| Hypothyroidism | 0.94 | [0.87, 1.01] | 0.93 | [0.86, 1.01] |
| Kidney failure/insufficiency | 1.00 | [0.89, 1.11] | 1.06 | [0.95, 1.18] |
| Liver disease | 1.12 | [0.81, 1.56] | 0.99 | [0.57, 1.72] |
| Peptic ulcer without bleeding | 3.45 | [0.99, 12.00] | 0.90 | [0.17, 4.87] |
| Congestive heart disease | 0.88 | [0.75, 1.03] | 1.01 | [0.86, 1.19] |
| Cardiac arrhythmia | 0.83*** | [0.75, 0.91] | 0.91* | [0.82, 1.00] |
| Heart valve disease | 0.78 | [0.60, 1.02] | 1.26* | [1.03, 1.53] |
| Pulmonary heart and diseases and of the pulmonary circulation | 0.69 | [0.36, 1.32] | 0.56* | [0.36, 0.88] |
| Peripheral vascular disease | 1.04 | [0.85, 1.27] | 1.00 | [0.82, 1.22] |
| High blood pressure without complications | 1.02 | [0.96, 1.10] | 1.04 | [0.96, 1.13] |
| High blood pressure with complications | 1.05 | [0.85, 1.30] | 0.97 | [0.78, 1.20] |
| Paralysis | 1.45 | [0.91, 2.31] | 1.36 | [0.71, 2.61] |
| Frailty-Score |  |  |  |  |
| low risk (<5) | (Reference) | | (Reference) | |
| intermediate risk (5 to 15) | 0.86 | [0.55, 1.35] | 0.92 | [0.67, 1.27] |
| Early rehabilitation complex treatment | 0.77 | [0.38, 1.53] | 0.85 | [0.53, 1.36] |
| Function-oriented physical therapy | 0.95 | [0.87, 1.02] | 0.93 | [0.85, 1.02] |
| Revision | 1.49 | [0.49, 4.50] | 2.34 | [0.79, 6.96] |
| Surgical complication | 1.13 | [0.79, 1.63] | 1.13 | [0.71, 1.81] |
| Femur fracture |  |  |  |  |
| General complication | 0.72* | [0.53, 0.99] | 0.95 | [0.66, 1.37] |
| Thrombosis/pulmonary embolism | 1.38 | [0.90, 2.11] | 1.28 | [0.79, 2.07] |
| Days in hospital |  |  |  |  |
| < 8 | (Reference) | | (Reference) | |
| 8 and 9 | 1.11** | [1.04, 1.18] | 1.01 | [0.93, 1.10] |
| > 9 | 1.22*** | [1.11, 1.33] | 1.09 | [0.99, 1.20] |
| **Pre-observation period (12 months)** | | | | |
| Osteoporosis | 1.02 | [0.93, 1.11] | 1.05 | [0.96, 1.15] |
| Atherosclerosis | 1.06 | [0.95, 1.17] | 0.98 | [0.88, 1.10] |
| Fibromyalgia | 1.31 | [0.77, 2.23] | 2.02 | [0.84, 4.84] |
| Segmental functional disorders | 1.03 | [0.95, 1.12] | 0.93 | [0.84, 1.02] |
| Anxiety disorders | 1.12* | [1.02, 1.23] | 0.94 | [0.81, 1.10] |
| Depression | 1.09* | [1.02, 1.16] | 1.07 | [0.97, 1.18] |
| Somatoform disorders | 1.20*** | [1.11, 1.29] | 1.15** | [1.04, 1.26] |
| Sleep disorder | 1.14*** | [1.06, 1.23] | 1.14** | [1.03, 1.25] |
| Mental and behavioral disorders caused by opioids: Dependence syndrome | 1.76 | [0.96, 3.24] | 1.34 | [0.54, 3.33] |
| Other neurotic disorders | 0.95 | [0.79, 1.14] | 1.10 | [0.86, 1.42] |
| Other personality and behavioral disorders: Development of physical symptoms | 1.15 | [0.44, 2.99] | 1.04 | [0.32, 3.41] |
| Consumption of alcohol, tobacco, medicines, or drugs | 1.00 | [0.57, 1.75] | 2.52 | [0.87, 7.33] |
| Gait and mobility disorders | 0.94 | [0.85, 1.04] | 1.04 | [0.96, 1.14] |
| Chronic pain syndrome with persistent personality change | 0.99 | [0.75, 1.31] | 1.09 | [0.70, 1.69] |
| Pain: psychogenic | 1.08 | [0.92, 1.27] | 0.85 | [0.68, 1.05] |
| Headache and migraine | 1.14** | [1.04, 1.25] | 1.05 | [0.90, 1.22] |
| Renal colic | 1.09 | [0.48, 2.50] | 2.63* | [1.12, 6.17] |
| Pain: abdomen | 0.96 | [0.81, 1.14] | 1.15 | [0.93, 1.42] |
| Pain: extremity | 1.22* | [1.02, 1.46] | 1.00 | [0.81, 1.25] |
| Pain: neck and thorax | 0.97 | [0.70, 1.34] | 0.88 | [0.59, 1.31] |
| Pain: spine | 1.10** | [1.03, 1.16] | 1.20*** | [1.10, 1.30] |
| Pain: ear | 0.91 | [0.49, 1.69] | 0.87 | [0.39, 1.91] |
| Complex regional pain syndrome | 1.37 | [0.77, 2.41] | 0.65 | [0.23, 1.82] |
| Other pain | 1.52*** | [1.43, 1.61] | 1.42*** | [1.32, 1.54] |
| Prescriptions prior to surgery | 7.73*** | [7.29, 8.18] | 7.15*** | [6.64, 7.70] |
| Received long-term analgesic | 1.01 | [0.92, 1.11] | 0.94 | [0.83, 1.07] |
| Received physiotherapy | 1.02 | [0.93, 1.11] | 1.05 | [0.96, 1.15] |
| **Process** | | | | |
| Days between hospital discharge and rehabilitation |  |  |  |  |
| < 3 days | (Reference) | | (Reference) | |
| 3 to 7 days | 1.00 | [0.92, 1.08] | 1.00 | [0.90, 1.11] |
| >7 days | 0.90** | [0.83, 0.97] | 0.91 | [0.81, 1.02] |
| Days in inpatient rehabilitation |  |  |  |  |
| < 8 | 1.39** | [1.10, 1.75] | 1.19 | [0.95, 1.49] |
| 8 to 21 | (Reference) | | (Reference) | |
| >21 | 1.19*** | [1.09, 1.29] | 1.10 | [0.99, 1.23] |
| Standardized complication rate (surgery hospital) |  |  |  |  |
| Low (<0.9) | (Reference) | | (Reference) | |
| Medium (0.9 to 1.1) | 1.00 | [0.93, 1.09] | 1.01 | [0.92, 1.11] |
| Higher (>1.1) | 1.06 | [0.98, 1.13] | 1.03 | [0.95, 1.12] |
|  |  |  |  |  |
| Intercept | 0.06*** | [0.05, 0.07] | 0.06*** | [0.05, 0.07] |
| *** p<.001, ** p<.01, * p<.05 |  |  |  |  |

Age was excluded as a risk factor because the stratification itself accounts for the age effect. Long-term analgesic prescription after arthroplasty is defined as a minimum of 20 DDD of analgesic prescription in each of 2 subsequent quarters (90-day periods). Outpatient ICD-10 diagnosis was defined as present if recorded in the database in at least 2 of 4 quarters (90d-period). Long-term analgesic use before surgery is defined as a minimum of 20 DDD of analgesic prescription in at least 3 of 4 quarters (90-day periods) in 365 days prior to surgery. Physical therapy is defined at least 1 prescription (Diseases of the extremities and pelvis) up to 90 days before surgery. BMI, Body Mass Index

**Table S6:** Sensitivity analyses: Full regression results including all evaluated risk factors from the logistic regression analyses for long-term analgesic prescription after hip or knee arthroplasty by excluding all patients with further surgeries on the musculoskeletal system during follow-up (180 days after rehabilitation)

|  | Hip arthroplasty  (n= 60743) | | Knee arthroplasty  (n= 69749) | |
| --- | --- | --- | --- | --- |
|  | OR | 95% CI | OR | 95% CI |
| **Demographics** | | | | |
| Age |  |  |  |  |
| below 64 | 1.30*** | [1.18, 1.43] | 1.36*** | [1.26, 1.47] |
| 65 to 69 | 1.04 | [0.96, 1.11] | 1.11** | [1.04, 1.18] |
| 70 to 74 | (Reference) | |  | |
| 75 to 79 | 0.98 | [0.90, 1.06] | 0.97 | [0.91, 1.04] |
| 80 to 84 | 0.95 | [0.88, 1.03] | 0.92* | [0.85, 0.99] |
| 85 and over | 0.87* | [0.77, 0.99] | 0.81** | [0.71, 0.92] |
| Gender |  |  |  |  |
| Male | (Reference) | | (Reference) | |
| Female | 1.12*** | [1.07, 1.19] | 1.03 | [0.97, 1.08] |
| Nursing care level |  |  |  |  |
| No care level | (Reference) | | (Reference) | |
| Care level 1 | 1.42*** | [1.30, 1.54] | 1.37*** | [1.26, 1.50] |
| Care level 2 | 1.66*** | [1.52, 1.81] | 1.57*** | [1.45, 1.69] |
| Care level 3 and higher | 2.10*** | [1.81, 2.45] | 1.79*** | [1.52, 2.10] |
| **Inpatient stay (arthroplasty)** | | | | |
| BMI 30-34 kg/m^2^ | 1.18*** | [1.09, 1.28] | 1.05 | [0.98, 1.12] |
| BMI 35-39 kg/m^2^ | 1.28*** | [1.16, 1.41] | 1.00 | [0.91, 1.09] |
| BMI 40+ kg/m^2^ | 1.40*** | [1.19, 1.64] | 1.29*** | [1.17, 1.42] |
| Alcohol abuse | 1.53 | [0.88, 2.66] | 1.11 | [0.62, 1.98] |
| Drug abuse | 1.50 | [0.69, 3.27] | 3.87** | [1.68, 8.92] |
| Psychosis | 0.59 | [0.33, 1.05] | 0.94 | [0.59, 1.51] |
| Depression | 1.26*** | [1.13, 1.39] | 1.05 | [0.96, 1.16] |
| AIDS/HIV | 2.56 | [0.12, 53.28] | 3.53 | [0.68, 18.35] |
| Rheumatic disease | 1.55*** | [1.38, 1.75] | 1.17** | [1.05, 1.30] |
| Coagulopathy | 1.02 | [0.80, 1.30] | 0.90 | [0.71, 1.14] |
| Weight loss | 1.01 | [0.76, 1.33] | 0.96 | [0.57, 1.63] |
| Disturbances of water, electrolyte or acid-base balance | 1.07 | [0.99, 1.17] | 1.07 | [0.98, 1.17] |
| Hemorrhagic anemia | 1.04 | [0.58, 1.86] | 0.82 | [0.36, 1.87] |
| Deficiency anemia | 1.11 | [0.88, 1.39] | 1.32* | [1.05, 1.67] |
| Other neurological disease | 1.01 | [0.84, 1.21] | 1.00 | [0.84, 1.19] |
| Chronic lung disease | 1.28*** | [1.18, 1.39] | 1.21*** | [1.12, 1.30] |
| Diabetes without complications | 1.09* | [1.02, 1.16] | 1.19*** | [1.12, 1.26] |
| Diabetes with complications | 1.23 | [1.00, 1.51] | 1.28** | [1.09, 1.51] |
| Hypothyroidism | 1.01 | [0.95, 1.06] | 0.94* | [0.88, 0.99] |
| Kidney failure/insufficiency | 1.02 | [0.93, 1.12] | 1.03 | [0.95, 1.11] |
| Liver disease | 0.89 | [0.66, 1.20] | 1.06 | [0.78, 1.45] |
| Peptic ulcer without bleeding | 1.70 | [0.44, 6.56] | 1.75 | [0.65, 4.71] |
| Congestive heart disease | 1.12 | [1.00, 1.25] | 0.99 | [0.88, 1.11] |
| Cardiac arrhythmia | 0.95 | [0.87, 1.02] | 0.87*** | [0.81, 0.94] |
| Heart valve disease | 0.94 | [0.79, 1.12] | 1.04 | [0.88, 1.24] |
| Pulmonary heart and diseases and of the pulmonary circulation | 0.84 | [0.56, 1.27] | 0.64* | [0.43, 0.96] |
| Peripheral vascular disease | 1.18* | [1.03, 1.34] | 1.05 | [0.90, 1.22] |
| High blood pressure without complications | 1.10** | [1.04, 1.16] | 1.04 | [0.98, 1.10] |
| High blood pressure with complications | 1.03 | [0.87, 1.21] | 0.99 | [0.85, 1.16] |
| Paralysis | 1.02 | [0.66, 1.57] | 1.34 | [0.89, 2.01] |
| Frailty-Score |  |  |  |  |
| low risk (<5) | (Reference) | | (Reference) | |
| intermediate risk (5 to 15) | 0.93 | [0.74, 1.17] | 0.91 | [0.69, 1.19] |
| Early rehabilitation complex treatment | 1.00 | [0.71, 1.41] |  |  |
| Function-oriented physical therapy | 1.00 | [0.94, 1.08] | 0.92 | [0.61, 1.37] |
| Revision | 0.95 | [0.55, 1.65] | 0.96 | [0.90, 1.03] |
| Surgical complication | 1.18 | [0.90, 1.56] | 1.62 | [0.70, 3.75] |
| Femur fracture | 1.25* | [1.00, 1.56] | 1.06 | [0.78, 1.42] |
| General complication | 0.90 | [0.73, 1.12] | 0.85 | [0.67, 1.09] |
| Thrombosis/pulmonary embolism | 1.06 | [0.55, 2.02] | 1.33 | [0.98, 1.82] |
| Days in hospital |  |  |  |  |
| < 8 | (Reference) | | (Reference) | |
| 8 and 9 | 1.05 | [1.00, 1.12] | 1.07** | [1.02, 1.13] |
| > 9 | 1.17*** | [1.09, 1.25] | 1.17*** | [1.09, 1.26] |
| **Pre-observation period (12 months)** | | | | |
| Osteoporosis | 1.09* | [1.01, 1.17] | 1.03 | [0.97, 1.11] |
| Atherosclerosis | 1.03 | [0.95, 1.11] | 1.04 | [0.96, 1.12] |
| Fibromyalgia | 1.01 | [0.52, 1.99] | 1.66* | [1.02, 2.71] |
| Segmental functional disorders | 0.99 | [0.92, 1.07] | 1.00 | [0.93, 1.06] |
| Anxiety disorders | 0.94 | [0.86, 1.03] | 1.03 | [0.94, 1.11] |
| Depression | 1.10** | [1.04, 1.17] | 1.07* | [1.01, 1.14] |
| Somatoform disorders | 1.23*** | [1.15, 1.31] | 1.18*** | [1.11, 1.26] |
| Sleep disorder | 1.22*** | [1.13, 1.32] | 1.15*** | [1.08, 1.23] |
| Mental and behavioral disorders caused by opioids: Dependence syndrome | 1.73* | [1.03, 2.90] | 1.81* | [1.02, 3.22] |
| Other neurotic disorders | 0.86 | [0.73, 1.03] | 0.97 | [0.83, 1.13] |
| Other personality and behavioral disorders: Development of physical symptoms | 0.65 | [0.28, 1.50] | 1.12 | [0.50, 2.48] |
| Consumption of alcohol, tobacco, medicines, or drugs | 0.89 | [0.46, 1.71] | 1.08 | [0.67, 1.73] |
| Gait and mobility disorders | 1.02 | [0.95, 1.10] | 1.01 | [0.94, 1.08] |
| Chronic pain syndrome with persistent personality change | 1.29 | [0.98, 1.70] | 0.95 | [0.75, 1.21] |
| Pain: psychogenic | 1.02 | [0.86, 1.19] | 0.95 | [0.83, 1.09] |
| Headache and migraine | 0.98 | [0.89, 1.09] | 1.10* | [1.01, 1.20] |
| Renal colic | 1.22 | [0.54, 2.76] | 1.77 | [0.99, 3.15] |
| Pain: abdomen | 1.08 | [0.94, 1.25] | 1.01 | [0.87, 1.18] |
| Pain: extremity | 1.13 | [0.97, 1.31] | 1.14 | [0.98, 1.32] |
| Pain: neck and thorax | 0.93 | [0.71, 1.23] | 0.94 | [0.72, 1.21] |
| Pain: spine | 1.17*** | [1.11, 1.24] | 1.15*** | [1.09, 1.21] |
| Pain: ear | 0.92 | [0.48, 1.73] | 0.88 | [0.52, 1.49] |
| Complex regional pain syndrome | 2.19** | [1.22, 3.93] | 1.05 | [0.64, 1.72] |
| Other pain | 1.55*** | [1.46, 1.64] | 1.48*** | [1.41, 1.56] |
| Prescriptions prior to surgery | 7.04*** | [6.71, 7.40] | 7.78*** | [7.42, 8.15] |
| Received long-term analgesic | 0.98 | [0.91, 1.07] | 0.97 | [0.89, 1.05] |
| Received physiotherapy | 1.09* | [1.01, 1.17] | 1.03 | [0.97, 1.11] |
| **Process** | | | | |
| Days between hospital discharge and rehabilitation |  |  |  |  |
| < 3 days | (Reference) | | (Reference) | |
| 3 to 7 days | 0.94 | [0.88, 1.01] | 0.98 | [0.91, 1.04] |
| >7 days | 0.90* | [0.84, 0.98] | 0.87*** | [0.82, 0.93] |
| Days in inpatient rehabilitation |  |  |  |  |
| < 8 | 1.27* | [1.06, 1.52] | 1.31** | [1.09, 1.57] |
| 8 to 21 | (Reference) | | (Reference) | |
| >21 | 1.18*** | [1.09, 1.29] | 1.14*** | [1.06, 1.22] |
| Standardized complication rate (surgery hospital) |  |  |  |  |
| Low (<0.9) | (Reference) | | (Reference) | |
| Medium (0.9 to 1.1) | 1.03 | [0.96, 1.10] | 1.00 | [0.94, 1.06] |
| Higher (>1.1) | 1.13*** | [1.06, 1.20] | 1.04 | [0.98, 1.10] |
|  |  |  |  |  |
| Intercept | 0.04*** | [0.04, 0.04] | 0.05*** | [0.05, 0.06] |
| *** p<.001, ** p<.01, * p<.05 |  |  |  |  |

Long-term analgesic prescription after arthroplasty is defined as a minimum of 20 DDD of analgesic prescription in each of 2 subsequent quarters (90-day periods). Outpatient ICD-10 diagnosis was defined as present if recorded in the database in at least 2 of 4 quarters (90d-period). Long-term analgesic use before surgery is defined as a minimum of 20 DDD of analgesic prescription in at least 3 of 4 quarters (90-day periods) in 365 days prior to surgery. Physical therapy is defined at least 1 prescription (Diseases of the extremities and pelvis) up to 90 days before surgery. BMI, Body Mass Index
